# Hepatic Insulin Resistance Model in the Male Wistar Rat Using Exogenous Insulin Glargine Administration

**DOI:** 10.3390/metabo13040572

**Published:** 2023-04-18

**Authors:** Victor Enrique Sarmiento-Ortega, Diana Moroni-González, Alfonso Diaz, Miguel Ángel García-González, Eduardo Brambila, Samuel Treviño

**Affiliations:** 1 Laboratory of Chemical-Clinical Investigations, Department of Clinical Chemistry, Meritorious Autonomous University of Puebla, 14 Sur. FCQ1, Ciudad Universitaria, Puebla City 72560, Mexico; 2 Department of Pharmacy, Faculty of Chemistry Science, Meritorious Autonomous University of Puebla, 22 South, FCQ9, Ciudad Universitaria, Puebla City 72560, Mexico; 3 Laboratory of Clinical Pharmacy, Faculty of Chemistry Science, Meritorious Autonomous University of Puebla, 22 South, FCQ10, Ciudad Universitaria, Puebla City 72560, Mexico

**Keywords:** insulin resistance, insulin signaling, inflammation, oxidative stress, dyslipidemia

## Abstract

Metabolic diseases are a worldwide health problem. Insulin resistance (IR) is their distinctive hallmark. For their study, animal models that provide reliable information are necessary, permitting the analysis of the cluster of abnormalities that conform to it, its progression, and time-dependent molecular modifications. We aimed to develop an IR model by exogenous insulin administration. The effective dose of insulin glargine to generate hyperinsulinemia but without hypoglycemia was established. Then, two groups (control and insulin) of male Wistar rats of 100 g weight were formed. The selected dose (4 U/kg) was administered for 15, 30, 45, and 60 days. Zoometry, a glucose tolerance test, insulin response, IR, and the serum lipid profile were assessed. We evaluated insulin signaling, glycogenesis and lipogenesis, redox balance, and inflammation in the liver. Results showed an impairment of glucose tolerance, dyslipidemia, hyperinsulinemia, and peripheral and time-dependent selective IR. At the hepatic level, insulin signaling was impaired, resulting in reduced hepatic glycogen levels and triglyceride accumulation, an increase in the ROS level with MAPK-ERK1/2 response, and mild pro-oxidative microenvironmental sustained by MT, GSH, and GR activity. Hepatic IR coincides with additions in MAPK-p38, NF-κB, and zoometric changes. In conclusion, daily insulin glargine administration generated a progressive IR model. At the hepatic level, the IR was combined with oxidative conditions but without inflammation.

## 1. Introduction

Diabetes prevalence has risen over the last three decades. According to the International Diabetes Federation, in 2021, there were 537 million cases of adult diabetes worldwide, which means that over 10.5% of the world’s adult population now have this condition [1]. Approximately 6.7 million deaths occur from diabetes or its comorbidities. The disease causes 9% of global healthcare expenditures, creating a heavy economic burden [2]. Particularly in type 2 diabetes (T2D), hyperglycemia is caused by chronically impaired insulin signaling, decreased insulin sensitivity, or insulin resistance (IR) [3]. IR is a distinctive hallmark of many metabolic diseases, such as metabolic syndrome, metabolic (dysfunction) associated fatty liver disease, polycystic ovary, cardiometabolic diseases, and even metabolic dementia [4,5,6]. However, although almost tissues possess insulin receptors and signaling pathways associated with the hormone (metabolic and mitogenic), not all develop IR simultaneously or with the same features [5]. Physiologically, IR is defined as an inability of some tissue to respond to normal levels of the hormone; thus, higher insulin concentration is required to maintain normal functions [7,8]. The main insulin actions are focused on glucose and lipid homeostasis, such as hepatic gluconeogenesis suppression, glycogen synthesis (liver and muscle), glucose uptake (muscle and adipose), lipogenesis (liver and adipose), and adipocyte lipolysis suppression [9].

Insulin signaling is mediated through insulin receptor tyrosine kinase. The conformational change results in the autophosphorylation of tyrosine residues and the subsequent activation of phosphotyrosine-binding proteins such as insulin receptor substrate (IRS) or SHC-transforming protein (Shc). Downstream, the metabolic pathway is sustained by IRS, phosphatidylinositol-3 kinase (PI3K), and protein kinase B (PKB/Akt). [9,10]. In the liver, Akt signaling represses gluconeogenesis and activates glycogen synthesis via glycogen synthase kinase 3β (GSK3β)-inhibition. It also activates lipid anabolism by upregulating sterol regulatory element-binding protein 1c (SREBP-1c), a master transcriptional regulator of hepatic de novo lipogenesis (DNL) [9]. In the mitogenic arm, insulin-activated Shc produces Ras/mitogen-activated protein kinase (Ras/MAPK) pathway activation. Ras interacts with IRS-1/2 and operates as a molecular switch, converting upstream tyrosine phosphorylation into a serine kinase cascade via the stepwise activation of Raf and the MAPKs MEK, ERK1, and ERK2. The MAPKs can initiate transcriptional programs that commit the cell to a proliferative or differentiative cycle and, in some cases, affect metabolic activity [11].

Oxidative stress and inflammation disrupt insulin’s metabolic and/or mitogenic pathways, developing and aggravating IR. It is well-known that oxidative stress induces inflammation and vice versa. Recent studies confirm the presence of oxidative stress biomarkers, such as malondialdehyde (MDA) and 4-hydroxyalkenal (4HDA), in cases of IR [8,12]. Therefore, hepatic redox balance is decisive in maintaining unaltered insulin signaling and metabolic functions. Likewise, inflammatory response characterized by altered cytokine production and the activations of inflammatory signaling pathways is actively investigated to determine its role in IR [13,14]. Inflammation-related cytokines, such as tumor necrosis factor-α (TNF-α), are mediated by the c-Jun N-terminal kinase (JNK) activity, which inhibits IRS and interferes with insulin receptor autophosphorylation and downstream insulin signaling as well [15,16]. TNF-α also activates the nuclear factor κ-B (NF-κB), increasing inflammatory cytokine expressions, such as interleukine-1β (IL-1β) and -6 (IL-6), which exacerbate IR [4]. However, it is unclear if oxidative stress precedes inflammation or vice versa.

Therefore, cellular or animal models are necessary for exploring IR pathogenesis and precise stages where oxidative stress and inflammation are developed. The cellular models are used to observe IR mechanisms, modulation or modification of signaling pathways, and the direct effects of intervening factors on IR [17,18]. However, they are limited because they do not offer complete information on compensation or decompensation mechanisms (comorbidities), such as in a whole organism. On the other hand, animal models offer different advantages and disadvantages. Experimental animal models provide an opportunity to study IR pathophysiology and its complications [17,19]. However, no single animal model presents all of these features that reflect human conditions. Researchers employ models of spontaneous IR, genetically modified and induced by chemical, pharmacological, or dietary means, but none allow us to study the progression of oxidative stress, inflammation, and metabolic defects associated with IR with precision or without methodological interferences. In this study, we aimed to develop an IR model by exogenous insulin administration that permits us to show the stages of hyperinsulinemia, loss of insulin sensibility, and early IR with their respective changes at the hepatic level of redox imbalance, inflammation, impairment of insulin signaling, and metabolic disorders.

## 2. Materials and Methods

### 2.1. Animals and Treatment

**Figure 1:**
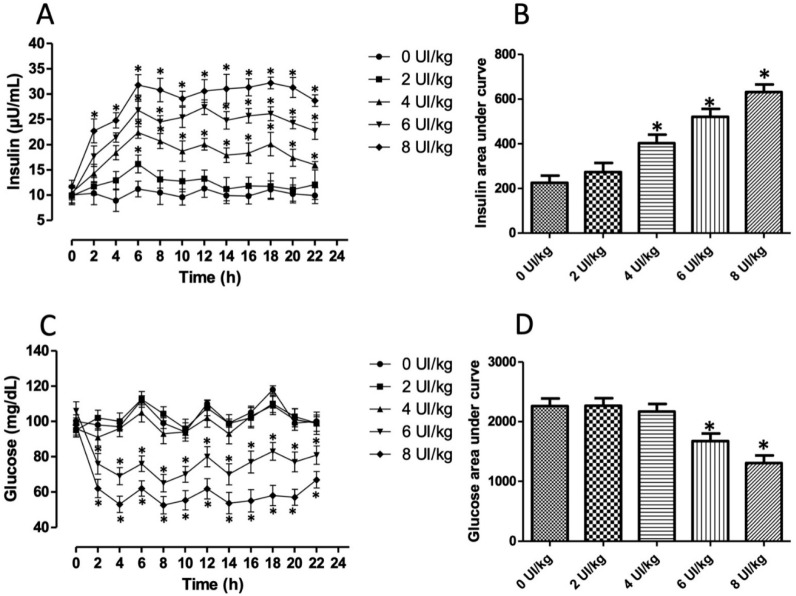
Effect of administering different doses of glargine on insulin and glucose concentration throughout the day. (**A**) Serum insulin concentration after insulin administration. (**B**) Area under curve of the insulin concentration. (**C**) Serum glucose concentration after insulin administration. (**D**) Area under curve of the glucose concentration. The results shown are the average of 10 different experiments ± SEM. (**A**,**C**) graphs were analyzed by a two-way ANOVA followed by a Bonferroni test. (**B**,**D**) graphs were analyzed by a One-way ANOVA followed by a Tukey’s post-test. (*) indicates significant difference regarding 0 UI/kg group, *p* ≤ 0.05.

One hundred thirty male Wistar rats (70–80 g) were provided from the vivarium “Claude Bernard” of the Universidad Autónoma de Puebla. Animals were preserved under controlled temperature conditions (22 °C) with 12 h of light and 12 h of darkness each cycle, with free demand for diet and water. The animals consumed a normocaloric food (5001, LabDiet; St. Louis, MO, USA) until they obtained weight of 100 g animals were assigned into two groups, (1) control group (n = 40) and (2) insulin group, with a daily subdermal administration of insulin glargine (4 U/kg = 146 µg/kg; n = 40). Lantus^®^ insulin glargine is a long-acting man-made insulin obtained from the rDNA of *Escherichia coli*. This dose was selected after administering different doses (0, 2, 4, 6, and 8 U/kg) of insulin glargine to ten rats per dose and measuring its effect on insulin and serum glucose concentration for 22 h, every 2 h (Figure 1). The dose of 4 U/kg was selected because it generated hyperinsulinemia without generating hypoglycemia. The insulin dose chosen was administered for 15, 30, 45, and 60 days (n = 10 per group). The CICUAL-BUAP ethics committee approved all the procedures described, and the guide for the Care and Use of Laboratory Animals of the Mexican Council for Animal Care NOM-062-ZOO-1999 was followed. These recommendations and some other national and international guidelines were followed for the best care of the animals.

### 2.2. Zoometry

Zoometric parameters such as weight, size, fat percentage, abdomen diameter, and body mass index (BMI) were evaluated at the end of different times in each experimental group as described elsewhere [20].

### 2.3. Oral Glucose Tolerance Test, Insulin Response, and Insulin Resistance Analyses

An oral glucose tolerance test (OGTT) was performed two days before finishing insulin glargine administration. The animals fasted for 4–5 h. A blood sample was obtained (0-time min) 4–5 h before collection. After oral administration of glucose (1.75 g/kg), blood samples were obtained from the tail vein at 30, 60, and 90 min, to later separate the serum by centrifugation (400× *g* for 10 min), and the samples were frozen at −70 °C until analysis. Glucose concentration (BioSystems, Guadalajara, Mexico) and insulin concentration (Diagnostica Internacional Company; Guadalajara, Mexico) were determined, and the area under the curve (AUC) was calculated [4]. The homeostatic model assessment insulin resistance (HOMA-IR), hepatic insulin sensitivity index (HIS), Matsuda–DeFronzo insulin resistance index, liver insulin resistance index (LIRI), and quantitative insulin sensitivity check index (QUICKI) were calculated as previously reported [21]. Other tissue-specific insulin sensitivity and resistance indexes were calculated to validate the model, as shown in Figure S1 [21].

### 2.4. Biochemical Assays

After the conclusion of the experimentation times, blood samples (700 μL) were taken from the tail vein under fasting conditions (4–5 h), and the serum was separated after centrifugation (400× *g* for 10 min). Triglycerides, high-density lipoprotein (HDL), and apoprotein B (ApoB) were determined using an A15 autoanalyzer (BioSystems, Guadalajara, Mexico). Very-low-density lipoprotein (VLDL) levels were estimated by the Martin-Hopkins equation [4]. As previously reported, the free fatty acid concentration (FFA) was determined [20]. 

### 2.5. Tissue for Biochemical, Immunoassay, and Histological Tests

After collecting blood samples, the rats were euthanized with sodium pentobarbital (75 mg/kg body weight, intraperitoneally). In all groups, the liver was excised. The major lobule was perfused with cold isotonic saline solution (SSI) and stored at −70 °C. The minor lobule was perfused with 10% formalin for histology. Procedures were performed as described in reference [4].

### 2.6. Biochemical Assays

Liver tissue (100 mg) was homogenized in 800 µL of SSI. In the homogenate, the concentration of triglycerides was quantified (BioSystems, Guadalajara, Mexico). For the concentration of glycogen, the Bennett method was used [22]. Brunk and Swanson’s method was used to quantify fatty acid (FA) [23]. All parameters were adjusted to 100 mg of tissue.

### 2.7. Redox Balance Assays

100 mg of liver tissue was homogenized in 700 mL of phosphate buffer saline (PBS) and centrifuged (2500× *g* for 30 min) at 4 °C in a 17 TR microcentrifuge. Total proteins were performed with the Sedmak and Grossberg method [24]. Reactive oxygen species (ROS) were determined using the 2′7′-dichlorodihydrofluorescein diacetate (DCFH-DA) method [4]. Nitrites were performed by using the Griess reaction. Results were expressed as micromoles of nitrite per milligram of protein (μM of NO_2_^−^/mg of protein) [4]. 

Lipoperoxidation products, malondialdehyde (MDA), and 4-hydroxyalkenal (4HDA) were measured in liver homogenate supernatants. To construct a standard absorbance curve to calculate the concentration of MDA + 4HDA [4], 0.5 to 5 μM of 1,1,3,3-tetra methoxy propane (10 mM stock) was used.

The metallothionein (MT) concentration was assayed by Eaton and Cherian protocol [25]. The enzymatic recycling technique determined the glutathione concentration. Total glutathione concentration, GSH, and GSSG concentration were calculated as described previously [4].

Glutathione peroxidase (GPx) activity was quantified with the Flohé and Günzler method [26]. Glutathione S-transferase (GST) activity was determined using the method described by Habig et al. [27]. Glutathione reductase (GR) activity was performed according to the method of Smith et al. [28].

The catalase activity (CAT) was quantified by Aebi methodology, and super oxide dismutase activity was described as previously [4].

### 2.8. Immunoassays

The liver tissue was homogenated with PBS and protease inhibitors at 4 °C to avoid degradation. The supernatant was obtained and used to determine IL-1β, IL-6, TNF-α, IL-10, TFG- β, and IL-1ar using ELISA commercial kits (Merck Millipore; Toluca, Mexico). Liver cytokine levels were reported as pg/mg protein. ELISA indirect was used for active forms of p-p38 (Tyr 182) and p-JNK (Thr 183 and Tyr 185); a standard protein concentration and 100 μL of 0.1 M carbonate buffer were placed into wells of ELISA and were incubated at 4 °C for 18 h. After three washes with PBS-Tween 20 (0.1%) solution, the plates were incubated (30 min) with bovine serum albumin (IgG free), then the plates were rewashed. The primary antibodies were incubated (2 h at room temperature) in each well. The plates were rewashed, and horseradish-peroxidase was added into the wells and incubated (2 h at room temperature). The antigen-antibody reaction was identified with 2,2′-azino-bis (3-ethylbenzthiazoline-6-sulfonic acid) in each well. The absorbance was determined after 15 min using a multiple benchmark reader at 415 nm (Bio-Rad; Hercules, CA, USA). The values were normalized according to the change in the control group.

### 2.9. Histological Assays

Blocks of liver tissue that were Paraffin-embedded were cut into 5-μm thick sections, paraffin was removed from these slides, and they were rehydrated for subsequent staining. Immunofluorescence and immunohistochemistry methodology were previously described [4]. The primary antibodies used were: p-insulin Rβ (Tyr 1361), p-Akt (Ser473), p-ERK1/2 (T202/Y204), Abcam Inc. (Toronto, Canada), SREBP-1c, NF-κB p65, ChEBP and p-GSK3β (Ser9) (Santa Cruz, CA, USA), p-insulin Rβ antibody (T1375), p-S6K1 (T389), and p-IRS (S307) from Merck Millipore (Toluca, Mexico). The secondary antibodies were: fluorescein isothiocyanate (FITC) and rhodamine red (Jackson ImmunoResearch Laboratories). Arbitrary units (pixels) were semi-quantified and normalized using the ImageJ program (National Institute of Health).

### 2.10. Statistical Analysis

A Shapiro–Wilk normality test was performed to verify that the different data come from a normally distributed population. The results were expressed as the mean ± SEM for all experiments. The results of the dose election, zoometry, and biochemical parameters were analyzed by a two-way ANOVA followed by a Bonferroni test. The AUC results for dose election were analyzed by a one-way ANOVA followed by a Tukey’s post-test. For quantitative variables compared with control groups in each time cohort, Student’s unpaired *t*-test was used. Finally, nonparametric variables were analyzed by the Mann–Whitney U test. Data analysis was performed with GraphPad Prism 5 (GraphPad Software Inc., USA). (*) The significance level was set at *p* ≤ 0.05.

## 3. Results

### 3.1. Hyperinsulinemia-Euglycemia Model

To choose an optimal dose of insulin glargine that generated hyperinsulinemia without hypoglycemia, we administered different doses ranging from 0 to 8 U/kg and quantified both parameters throughout 22 h, every 2 h. Serum insulin concentration significantly increased according to the dose administered (*p* < 0.0001; F = 200.6; Figure 1A). The group administered only with vehicle (0 U/kg; line with circle) showed an insulin variation during the day from 6 to 12 µU/mL with slight variations that corresponded to time feeding. All groups administered with exogenous insulin showed a positive slope that reached its maximum peak at 6 h. Depending on the dose (4 U/kg to up), high insulin concentrations were maintained for 22 h. This behavior was similar to that reported by the manufacturer. Higher doses (6 and 8 U/kg) increased from two hours. The AUC significantly increased in dosages of 4 U/kg (78%), 6 U/kg (130%), and 8 U/kg (179%) regarding the 0 U/kg group (*p* < 0.0001; Figure 1B). On the other hand, glucose concentration significantly decreased in the groups administered with 6 and 8 U/kg of insulin glargine (*p* < 0.0001; F = 178.8; Figure 1C). Mild hypoglycemia was observed along time analysis in the group administered with 6 U/kg of insulin; severe hypoglycemia was recorded in rats administered with 8 U/kg. The AUC decreased by 26% and 42% in these groups (*p* < 0.001; Figure 1D). Therefore, we chose the dose of 4 U/kg, which generated hyperinsulinemia without hypoglycemia to induce experimental insulin resistance.

### 3.2. Insulin Resistance Model

Insulin resistance is associated with impairment in zoometry, glucose homeostasis, and chronic hyperinsulinemia (Table 1). Weight and size were unaffected. Meanwhile, at 60 days of glargine administration (4 U/kg/day), BMI and fat percentage increased by 8% (*p* < 0.05) and 34% (*p* < 0.001). In each cohort time, an OGTT was performed. Glucose and insulin were measured at fasting and 30, 60, and 90 min after OGTT. After 15 days of glargine administration, fasting and postprandial glucose concentrations at 90 min were more significant than the control group, 17.8% and 11.8% (*p* < 0.05), while insulin concentration was not different. At 30 days, OGTT in the insulin group was significantly increased (*p* < 0.001) in fasting (25.8%), at 60 min (65.2%) and 90 min (30.4%), with hyperinsulinemia at 60 and 90 min, 66% and 144.6% (*p* < 0.0001). At 45 days, glucose tolerance worsened (*p* < 0.0001) in fasting (35.7%), at 30 min (40.7%), 60 min (23.4%), and 90 min (50%); and insulin response at 60 and 90 min, 114% and 41.2% (*p* < 0.0001). At 60 days, glucose tolerance was impaired (*p* < 0.0001) in fasting (19.7%), at 30 min (20%), 60 min (20%), and 90 min (64%), while significant hyperinsulinemia was observed in the times analyzed by 25.8%, 103.4%, 194%, and 131% (*p* < 0.0001). The AUC of OGTT and insulin response increased from 30 days by 38.2% and 47% (*p* < 0.01, *p* > 0.001), at 45 days by 34.8% and 15.3% (*p* < 0.01, *p* > 0.05), and at 60 days by 37.3% and 76.2% (*p* < 0.01, *p* > 0.0001). To investigate insulin resistance, we evaluated the HOMA-IR index, which showed a significant increase after 30, 45, and 60 days of glargine administration by 62%, 38%, and 61% (*p* < 0.001). Meanwhile, insulin sensitivity evaluated using QUICKY and Matsuda–DeFronzo indexes decreased by 22% and 30% (30 days; *p* < 0.01), 21% and 39% (45 days; *p* < 0.01), and 11% and 44% (60 days; *p* < 0.001). Specific hepatic insulin resistance was evaluated by the LIRI index, increasing at 45 and 60 days by 62% and 105% (*p* < 0.0001). However, the sensitivity in this tissue diminished from 15 days to the end of the study by 36%, 38%, 26%, and 38% (HIS index; *p* < 0.001).

### 3.3. Glargine Administration Impairs Hepatic Insulin Metabolic Signaling

**Figure 2:**
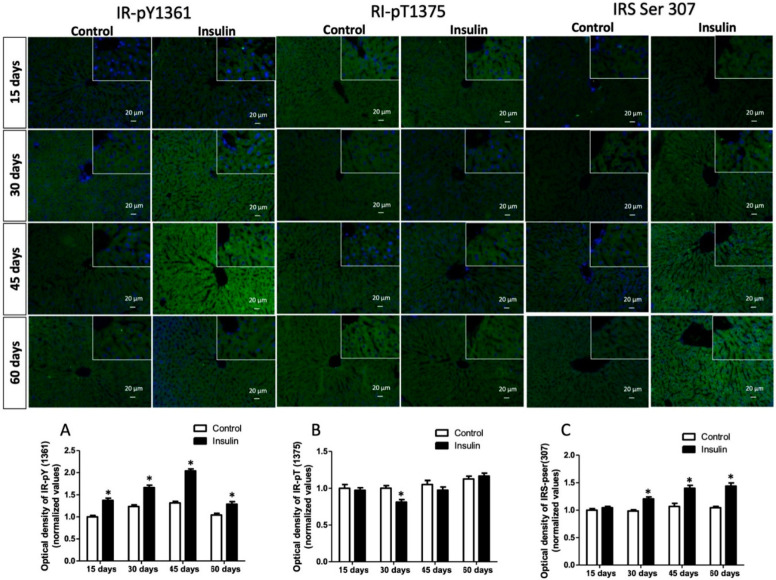
Evaluation of phosphorylation dynamic in the insulin receptor and IRS. (**A**) Tyrosine phosphorylation of insulin receptor (Y1361); (**B**) threonine phosphorylation of insulin receptor (T1375); (**C**) IRS serine phosphorylation of substrate insulin receptor (S307). Results are the mean average of 10 separate experimental animals per group ± SEM. (*) indicates a significant difference regarding control groups (*p* ≤ 0.05 by Mann–Whitney U test). The scale bar corresponds to 20 µm in a magnification of 400×.

**Figure 3:**
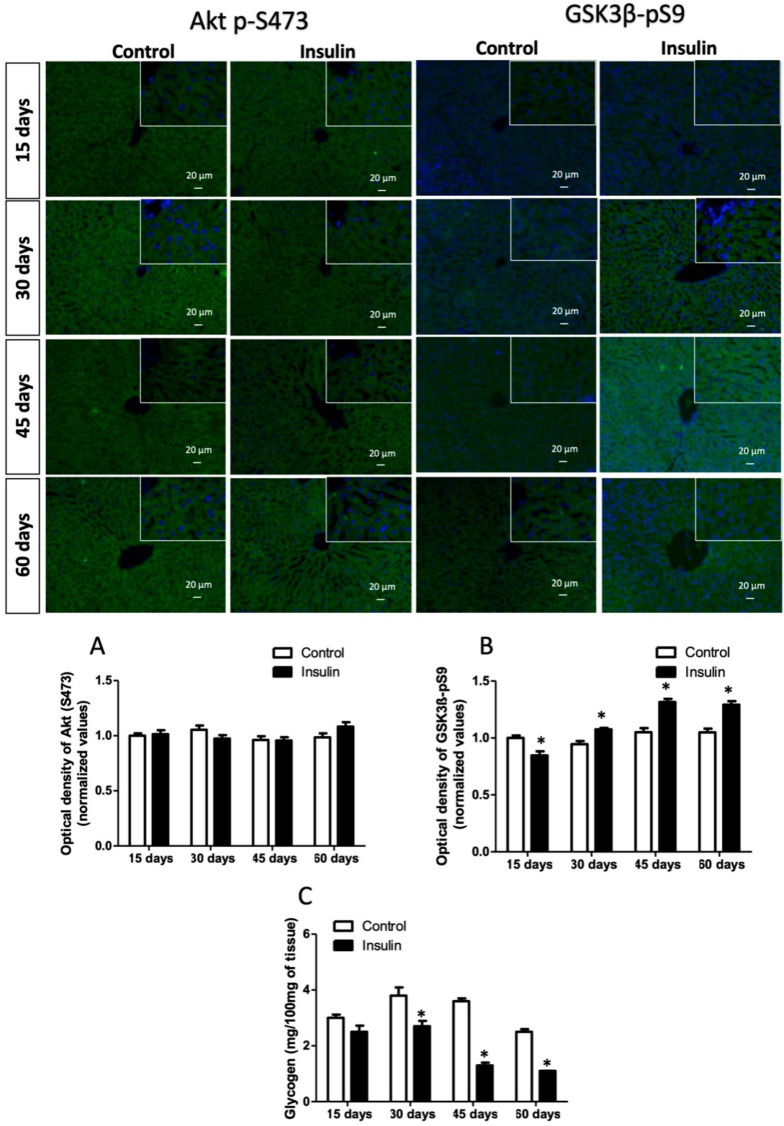
Evaluation of the hepatic glycogen pathway. (**A**) Serine phosphorylation of Akt (S473); (**B**) serine phosphorylation of GSK3β (S9); (**C**) hepatic glycogen concentration. Results are the mean average of 10 separate experimental animals per group ± SEM. (*) indicates a significant difference compared to the control groups (*p* ≤ 0.05). The optical density of figures (**A**,**B**) was analyzed with the Mann–Whitney U test, while the Student’s *t*-test analyzed glycogen (**C**). The scale bar corresponds to 20 µm in a magnification of 400×.

Due to insulin resistance modifying the signaling pathway, we analyzed its metabolic arm in the liver. First, we analyzed the immunoreactivity insulin receptor phosphorylated in tyrosine, which was significantly increased by 37%, 34%, 54%, and 23% at 15, 30, 45, and 60 days regarding control groups (Figure 2A; *p* < 0.05). In addition, the immunoreactivity of the insulin receptor phosphorylated in threonine was evaluated, and results showed only a decrease of 18% at 15 days (Figure 2B; *p* < 0.05). The next step in insulin signaling corresponds to IRS, which significantly increased in groups where glargine was administered for 30 days (22%), 45 days (20%), and 60 days (20%) in vehicle groups (Figure 2C; *p* < 0.05). Akt immunoreactivity did not show a difference between groups (Figure 3A; *p* = 1.0). However, GSK3β interestingly decreased by 23% at 15 days but increased by 15% at 30 days, 51% at 45 days, and 46% at 60 days (Figure 2B; *p* < 0.05). This branch of the pathway ends with glycogen synthesis, which decreased by 16%, 28%, 63%, and 56% in the times analyzed (Figure 3C; *p* < 0.05).

**Figure 4:**
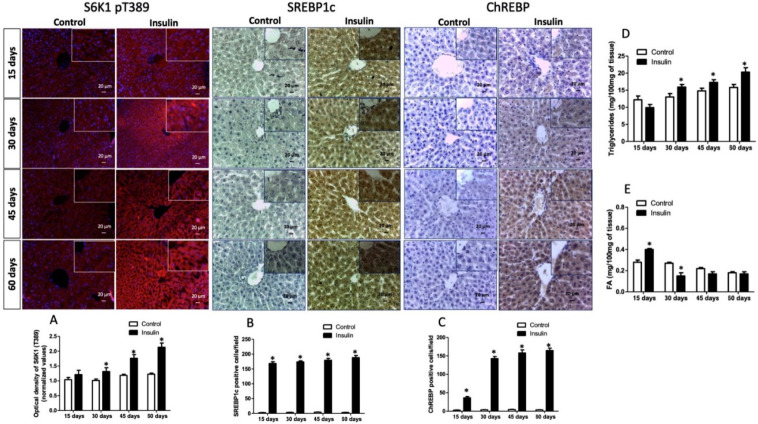
De novo lipogenesis pathway analysis. (**A**) Threonine phosphorylation of S6K1 (T389); (**B**) number of immunoreactive cells for SREBP1c; (**C**) number of immunoreactive cells for ChREBP; (**D**) hepatic triglyceride concentration; (**E**) hepatic fatty acid concentration. Results are the mean average of 10 separate experimental animals per group ± SEM. (*) indicates a significant difference compared to the control groups (*p* ≤ 0.05). Figures (**A**–**C**) were analyzed by Mann–Whitney U test, and (**D**,**E**) figures were analyzed by the Student’s *t*-test. The scale bar corresponds to 20 µm in a magnification of 400×.

DNL is a derivative Akt signaling branch. Therefore, we also analyzed the S6K1 immunoreactivity, which progressively increased from 30 days (29%), 45 days (48%), and 60 days (85%) regarding control groups (Figure 4A; *p*< 0.001). In addition, the number of immunoreactive cells per field for SREBP1c increased between 160- to 180-fold from 15- to 60 days in rats administered with glargine (Figure 4B; *p* < 0.0001). ChREBP is additive to hepatic DNL; the number of immunoreactive cells in groups administered with glargine also increased from 15 days to the end of the experiment by 45-fold (at 15 days), 140-fold (at 30 days), 155-fold (at 45 days), and 170-fold (at 60 days) (Figure 4C; *p* < 0.0001). DNL increases the biosynthesis and storage of FA and triglycerides in hepatocytes. FA increased at 15 days (42%; *p* < 0.01) and decreased by 44% (at 30 days; *p* < 0.01) and 22% (at 45 days; *p* < 0.05) (Figure 4E). Meanwhile, triglycerides decreased by 34% at 15 days (*p* < 0.05) and increased by 13% at 30 days, 16% at 45 days and 28% at 60 days (Figure 4D; *p* < 0.05).

### 3.4. Effect of Glargine Administration on Serum Lipid Profile

Variation in the lipid dynamic was observed. Interestingly, at 15 and 30 days of glargine administration, significantly diminished serum triglycerides (33% and 28%; *p* = 0.0391) and VLDL (33% and 28%; *p* = 0.0391) were observed, without ApoB changes. However, at 45 and 60 days, both parameters increased by 25% and 26% (*p* = 0.0409) and 16% and 23% (*p* = 0.0431), respectively, while ApoB concentration was augmented by 20% at 60 days of treatment (*p* = 0.0299). Additionally, HDL decreased by 23% (at 15 days), 15% (at 30 days), 9% (at 45 days), and 15% (at 60 days) (*p* = 0.0256), while FFA increased by 33%, 27%, 8%, and 19% in the insulin groups at the same time (*p* = 0.0312) (Table 2).

### 3.5. Effect of Insulin Glargine Administration on Hepatic Redox Balance and MAPK Response

**Figure 5:**
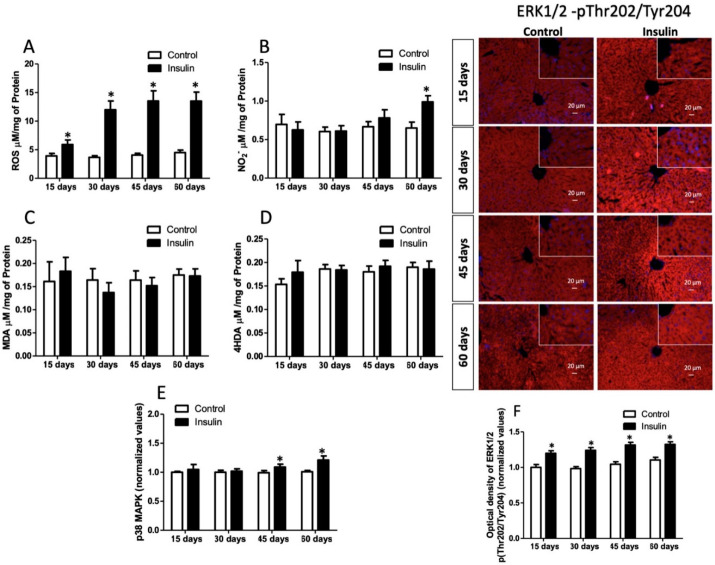
Hepatic oxidative stress and MAPK response. (**A**) Reactive oxygen species (ROS); (**B**) nitrites (NO_2_^−^); (**C**) malondialdehyde (MDA); (**D**) 4-hydroxyalkenal (4HDA); (**E**) p38-MAPK level; (**F**) ERK1/2-MAPK immunoreactivity. (*) indicates a significant difference compared to the control groups (*p* ≤ 0.05). Figures (**A**–**D**) were analyzed by the Student’s *t*-test, and figures (**E**,**F**) were analyzed by the Mann–Whitney U test. The scale bar corresponds to 20 µm in a magnification of 400×.

Oxidative stress is associated with insulin signaling loss. Thus, we evaluated hepatic redox balance and MAPK response. Results showed a significative ROS concentration increase of 50% (15 days), 225% (30 days), 231% (45 days), and 200% (60 days) compared to control groups (Figure 5A; *p* = 0.026). Nitrite concentration only increased by 41% at 60 days of glargine administration (Figure 5B; *p* = 0.041). Meanwhile, lipid peroxidation [(MDA; *p* = 0.6672) and (4HDA; *p* = 0.3392)] showed no difference between the groups (Figure 5C,D). Insulin resistance can develop through the MAPK-overactivated pathway. Hence, we evaluated the immunoreactivity of p38-MAPK, which increased by 9% and 20% at 45 and 60 days (Figure 5E; *p* = 0.0294). Meanwhile, ERK 1/2 immunoreactivity increased by 19%, 26%, 26%, and 19% at 15, 30, 45, and 60 days in insulin glargine-administered groups (Figure 5F; *p* = 0.0286).

Additionally, we evaluated hepatic antioxidant defense. The GSH concentration was reduced by 80% at 15 days, 78% at 30 days, and 58% at 45 days, while at 60 days, it was restored (*p* = 0.0311). In the insulin groups, oxidized glutathione species, GSSG, increased by 138%, 101%, 70%, and 58% at 15, 30, 45, and 60 days (*p* = 0.0207). However, total glutathione concentration showed no significant changes. Therefore, the redox index (2GSH/GSSG) was lowered at 15 days (93%), 30 days (90%), 45 days (85%), and 60 days (47%). MT concentration increased by 30% (at 15 days), 92% (at 30 days), 42% (at 45 days), and 53% (at 60 days) (*p* = 0.0322). Meanwhile, GPx and GT activity was not different between groups, but the GR activity increased by 53%, 54%, 57%, and 64% after 15, 30, 45, and 60 days of treatment. Finally, CAT and SOD activity did not differ between groups (Table 3).

### 3.6. Insulin Glargine Administration on the Hepatic Inflammation

**Figure 6:**
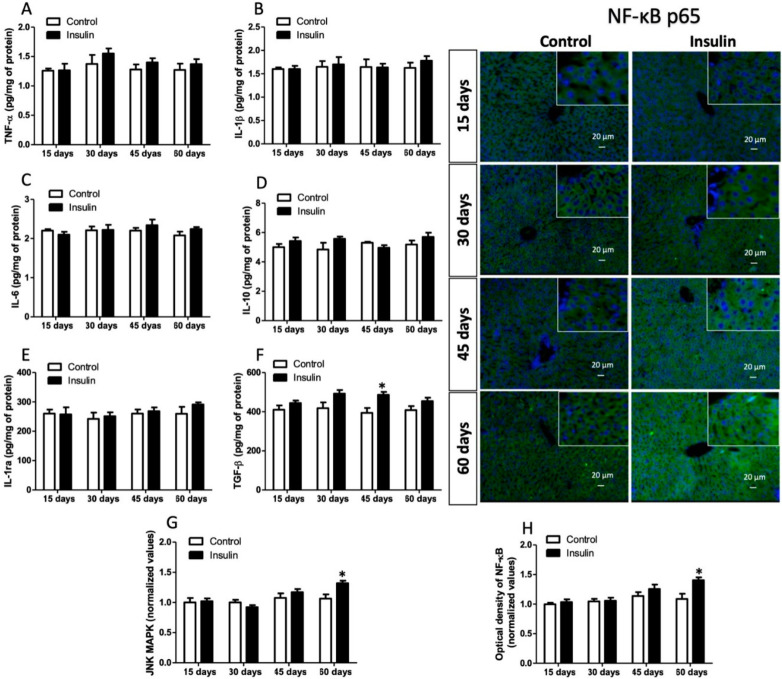
Hepatic inflammation status. (**A**) TNF-α; (**B**) IL-1β; (**C**) IL-6; (**D**) IL-10; (**E**) IL-1Ra; (**F**) TGF-β; (**G**) JNK-MAPK level; (**H**) NF-κB (p65) immunoreactivity. Results are the mean average of 10 separate experimental animals per group ± SEM. (*) Indicates a significant difference compared to the control groups (*p* ≤ 0.05). Figures (**A**–**F**) were analyzed by the Student’s *t*-test, and figures (**G**,**H**) were analyzed by the Mann–Whitney U test. The scale bar corresponds to 20 µm in a magnification of 400×.

Insulin resistance development is also associated with inflammation. Therefore, we evaluated pro-inflammatory and anti-inflammatory cytokines. Pro-inflammatory cytokines TNF-α, IL-1B, and IL-6 showed no changes in the time cohort analyzed. Likewise, anti-inflammatory interleukins IL-10 and IL-1ra had the same behavior. However, in the insulin group, TGF-β only increased by 26% at 45 days (Figure 6F; *p* < 0.05). Meanwhile, positive inducers such as JNK-MAPK and NF-κB increased at 60 days by 24% and 23% (Figure 6G,H; *p* < 0.05).

## 4. Discussion

Rodents, especially rats and mice, are the most widely used preclinical animal models to study metabolic disorders because their physiology is closer to humans. Hence, we developed an IR model using exogenous insulin administration to follow the stages of hyperinsulinemia, loss of insulin sensibility, and early IR. Our results showed that a subdermal dose of insulin glargine of 4 U/kg generated hyperinsulinemia without hypoglycemia for 22 h (Figure 1). Glucose and insulin AUC were used to choose the effective dose. Insulin glargine is an insulin analog of prolonged action (18–26 h). Glycine substitution for asparagine (A-chain, position 21) and two arginine residues addition (B-chain, position 30) is the reason for its prolonged time action. The structural changes render glargine precipitates forming hexamers at physiological pH after injection into the subcutaneous space, dissociating in active monomers slowly absorbed into the circulation [29]. Glargine properties result in a prolonged action with a modest peak of hyperinsulinemia dose depended, as shown in our results, indicating its lasting bioavailability. Similar results were reported by Juan et al., where chronic hyperinsulinemia induced with human insulin (1 U/d) released from subcutaneously implanted minipumps developed IR after ten days [30]. Exogenous insulin administration can also influence hepatic oxidative stress and inflammation because insulin mitogenic signaling (MAPK pathway) is intimately linked; thus, IR can be developed [31].

Reduced hepatic insulin sensitivity and glucose homeostasis without IR was observed after 15 days of exogenous insulin administration. At 30 days of evaluation, there was also a diminished glucose tolerance with hyperinsulinemia and systemic IR, but no hepatic IR was shown. Notably, from 30 days and adipose and cardiovascular IR and reduced muscle insulin sensibility were observed (S1). Consequently, at 45 days, hepatic IR was evidenced, and at 60 days zoometric changes in BMI and body fat percentage (Table 1) were presented. Results evidenced progressive metabolic and zoometric changes associated with insulin signaling impairment. Although the hyperinsulinemic-euglycemic clamp is the gold standard for IR measurement, several mathematical models are clinically useful surrogate IR measures, including HOMA-IR, Matusda–DeFronzo index, QUICKI, HIS, and LIRI, which have been validated for humans and rodents [4,5,13,21,32]. Additionally, analyzing serum glucose and insulin response to a glucose challenge helps establish a deteriorated metabolism. Fasting and postprandial glucose impairment are hallmarks of selective hepatic insulin resistance because deteriorating insulin signaling affects gluconeogenesis and glycogen synthesis [33,34]. The underlying mechanism has yet to be fully established, but the hypothesis on modifications in substrate specificities of Akt phosphorylation that affect gluconeogenesis and glycogenesis pathways is frequently studied. Therefore, we evaluated the insulin pathway.

Exogenous insulin administration caused an increase in the tyrosine (1361) phosphorylation of insulin receptors from 15 days and in serine (307) of IRS from 30 days to the end of the study (Figure 2A,C). The IRS phosphorylation leads to the activation of PI3K and, subsequently, of Akt. However, it showed no changes; a high immunoreactivity of GSK3β in Ser9 was observed (Figure 3B), which, together with IRS, suggests that this node of insulin signaling was strongly activated. Consequently, hepatic glycogen concentration was progressively diminished from 30 days of insulin administration (Figure 3C). It has been reported that Akt Ser473 phosphorylation may activate some signaling nodes related to gluconeogenesis, such as FOXO [9,35]. These activations might be suppressed in an IR status, which in turn causes plasma glucose to increase. At the same time, there was a reduced capacity for synthesis and storage of hepatic glycogen, resulting in progressive hyperglycemia both in fasting and postprandial, as was observed in our results. Several studies have shown that excessive postprandial glucose release (caused by IR) into the circulation has been uniformly found in patients with impaired glucose tolerance and T2D [35,36,37,38,39].

Another possible mechanism of selective hepatic IR involves insulin-induced SERBP-1c activation that modulates DNL. The balance between lipogenesis activity and gluconeogenesis suppression requires specific insulin levels and fine control at the signaling level. Elevated Akt activity in the early postprandial stage reduces hepatic glucose production, while Akt activity in the late stage increases the DNL [36,40]. Therefore, unterminated prolonged Akt activity enhances DNL and does not suppress gluconeogenesis. In the liver, DNL is organized by mTORC1 through insulin-induced Akt, where excess glycolytic products are converted to fatty acids via SREBP1c and S6K1. S6K1 acts as a downstream effector of mTORC1 that activates SREBP1c [41,42]. Our results showed that S6K1 increased after 30 days of insulin administration, while SREBP1c overexpression occurred after 15 days (Figure 4A,B), suggesting that insulin signaling reprogramming has occurred because of reduced insulin sensitivity, aggravated by advanced IR status. Lipogenesis is also mediated by the carbohydrate response element-binding protein (ChREBP) and liver X receptor-mediated SREBP1c, and these alternative lipogenesis pathways are activated by monosaccharides [43,44]. Our results also showed an increase of positive cells for ChREBP from 15 days of insulin administration. Hepatic ChREBP overexpression induces a fatty liver in mice and humans. ChREBP correlates with metabolic dysfunction associated with hepatosteatosis and steatohepatitis in humans [45]. Therefore, its deletion reduces fatty acid synthesis independently of SREBP1c [46]. ChREBP knockdown reduces liver DNL and hepatosteatosis in ob/ob mice [47]. Although it is now well established that DNL, metabolic dysfunction associated with hepatosteatosis, and steatohepatitis are increased in liver insulin-resistant people [48,49], the precise activation status of SREPB1c and ChREBP remains to be determined.

In addition, our results showed that the liver increased FA but not triglyceride concentration at the first 15 days of exogenous insulin administration (Figure 4D,E). However, triglycerides were significantly diminished in serum, but FFA increased (Table 2), suggesting hepatic DNL and triglyceride mobilization into circulation. At the same time, muscle and adipose clearance decrease plasma triglyceride concentration. However, fatty tissue cannot store them and returns to plasma as FFA because of a possible effect of adipocyte insulin resistance. Consequently, hepatic FFA uptake increases, causing a vicious lipogenic cycle. After 30 days, the hepatic phenotype changed because triglyceride storage progressively augmented (Figure 4D). Insulin signaling determines the balance of lipid secretion because it acts as a negative regulator of VLDL, triglyceride, and ApoB secretion [50]. Insulin concentrations modulate the expression and activity of microsomal triglyceride transfer protein, translation, and degradation of ApoB [51]. Physiologically, these insulin effects temporarily suppress lipid output from the liver, allowing efficient disposal of fat in peripheral organs in the postprandial state. Hepatic IR increases basal VLDL production, and insulin-dependent suppression of VLDL secretion is impaired [52,53]. In addition, newly generated and uptake fatty acids can undergo various biological modifications, including desaturation, elongation, and esterification, before being stored as triglycerides or exported as VLDL1 particles [48,54]. Hepatic IR modifies the VLDL phenotype, changing small VLDLs (VLDL2) with large VLDLs (VLDL1), which slows hepatic triglyceride clearance, increasing hepatic steatosis and causing hypertriglyceridemia, as our results showed after 45 days of treatment [5,55]. Moreover, hepatic low insulin sensitivity and IR also diminished HDL from 15 days of treatment. Numerous animal models support the notion that IR is caused by ectopic lipid accumulation in the liver and vice versa. Hepatic lipid accumulation is caused by a short-term high-fat diet, high-carbohydrates diet feeding, or lipid infusions, which induce IR in rats [5,56]. In addition, overexpression of hepatic lipoprotein lipase induces peripheral IR and lipid accumulation [51,57]. Furthermore, models of liver-specific knockdown of fatty acid transporter protein 2 (FATP2) or FATP5 reduce HFD-induced hepatosteatosis and increased glucose tolerance [58,59]. Therefore, our model can also study early molecular mechanisms and dynamics of hepatic steatosis associated with IR.

Insulin resistance and hepatic steatosis have been related to oxidative stress development. ROS at a physiological concentration positively influences intermediaries of insulin signaling. However, ROS excess leads to oxidative damage before and during the development of IR in vivo and in vitro [60,61]. Even though mitochondria are the main site of ROS production involving excessive nutrient fluxes, stressors like cytokines and FFAs also interfere with the physiological action of insulin in the liver [61]. It has been reported that lipid-induced endoplasmic reticulum stress [62,63], when unresolved, contributes to developing insulin resistance, inflammation, and cell death associated with fatty liver [64]. Our model also showed that ROS levels increased with hyperinsulinemia (at 15 days) before and during IR development (Figure 5A). ROS can activate the MAPK—ERK, an extracellular signal-regulated kinase pathway. MAPK pathway is part of the mitogenic arm of insulin signaling, which acts as a counterregulatory of metabolic signaling (minutes to hours) by a negative-feedback loop of insulin action by phosphorylation of IRS-1 on serine residues [9,65]; however, when it is found to be chronically active (days, months, or years), it is the major factor in developing IR [9]. This signaling controls diverse cellular functions, such as growth, differentiation, apoptosis, and proliferation [66]. ERK1/2 senses oxidative stress levels, acting as a switch that turns on cell survival mechanisms. Our results showed an increase in immunoreactivity phosphorylation (T202/Y204) from 15 days of analysis, which indicate an ERK1/2 activity that coincides with ROS levels (Figure 5F). Additionally, other MAPK, p38, a relevant protein that is activated in high oxidative stress levels, preventing cell damage via p53. p53 has pleiotropic actions for cell protection; metabolically, it regulates fatty acid metabolism, increasing fatty acid oxidation, triglyceride synthesis, and oxidative phosphorylation energy pathways [67,68]. In addition, p53 activated via MAPK-p38 enhances Nrf2 and reduces the effects of ROS/RNS and cellular senescence while diminishing glycolytic and apoptotic signaling [69]. Our results showed that MAPK-p38 increases at the same time that hepatic IR appears (at 45 days of evaluation; Figure 5E), while NO_2_^−^, a biomarker of RNS, only increases at 60 days (Figure 5B).

Interestingly, lipid peroxides MDA and 4HDA, hallmarks of oxidative stress, were no different between groups (Figure 5C,D). Thus, we evaluated the antioxidant defense to understand redox balance. GSH and MT can bind to lipid peroxides and other oxidative molecules, lowering their toxicity [4,70]. GSH and MT are the main non-enzymatic cell defense. MT increased during the study, while GSH diminished from 15 to 45 days. GSSH levels and redox index indicate that the microenvironmental was pro-oxidative every time it was analyzed. However, GR activity was increased according to GSH and GSSG concentration because this enzyme maintains GSH levels. GPx, GT, SOD, and CAT activity were not different between groups (Table 3). Contradictory results of redox balance have been reported in animal models and humans, possibly because antioxidant defense depends on metabolic disorder grade, time duration, comorbidities, the number of tissues involved, and mitochondrial defects [71,72,73,74]. In the present study, we studied the early stages of impaired insulin signaling, and thereby results showed no severe oxidative deterioration. However, ROS can activate MAPK-JNK, which in turn leads to IR.

MAPK-JNK can also induce IR via IRS phosphorylation (Ser 307). Our results showed a JNK increase at 60 days of insulin administration (Figure 6G). Likewise, in the early stage, JNK induces an inflammatory response through TNF-α. TNF-α, in turn, activates the inhibitor of nuclear factor κ-B kinase (IKK), which protects hepatocytes against IR, improves hepatic insulin signaling, and reduces inflammatory cytokine expression [75,76]. Therefore, TNF-α and NF-κB play a key role in developing inflammatory conditions impairing insulin signaling and generating IR. TNF-α results showed no changes, while NF-κB immunoreactivity increased at 60 days of analyses according to JNK levels (Figure 6A,H). However, pro-inflammatory cytokines (IL-6 and IL-1β) did not differ between groups (Figure 6B,C). Liver-specific knockout of NF-κB essential modulator improves fasting plasma glucose and insulin, glucose tolerance, and anti-inflammation in the liver [77]. In addition, in the early stage, the NF-κB overexpression (p65 subunit) somewhat protects against diet-induced IR and improves hepatic insulin sensitivity [78]. Furthermore, increased plasma levels of pro-inflammatory cytokines, such as TNF-α and IL-6, have been reported in people with severe IR, metabolic syndrome, T2D, and age-induced IR in elderly subjects [79,80,81]. These studies have demonstrated that chronic inflammation is not a primary causative factor of IR and is insufficient to disrupt glucose metabolism. Anti-inflammatory cytokines such as IL-10 and IL-1ra were not different between experimental groups (Figure 6D,E). However, TGF-β showed a mild increase (significative at 45 days; Figure 6F). Lipid accumulation in hepatocytes, endoplasmic reticulum (ER) stress, and ROS activate fibrotic pathways through TGF-β [82]. TGF-β induces hepatic stellate cell activation, resulting in the expression of α-smooth muscle actin and S100 calcium-binding protein A6, then the formation of stress fibers and the deposit of extracellular matrix components [45]. Chronically, pro-fibrotic conditions are a risk factor for developing severe IR, metabolic-associated fatty liver disease, and steatohepatitis. Thus, chronic inflammation indirectly exacerbates insulin resistance and should not be considered a primary strategic target for treatment. Our results suggest that inflammation is established in the late stages of IR.

Numerous experimental pieces of evidence have highlighted a link between insulin resistance, oxidative stress, and inflammation. However, how the apparition is ordered is still being determined. Our model shows that exogenous insulin administration initially causes fasting and postprandial hyperglycemia, associated with ROS overproduction without hepatic inflammation. The mechanisms could be related to an appropriate antioxidant response and adequation of the MAPK pathway, preventing NF-κB activity and, consequently, inflammatory cytokine release. The chronicity of hyperinsulinemia deteriorates hepatic response; thereby, selective insulin resistance appears. At the end of the experiment, JNK and NF-κB increased. Thus, under our experimental conditions, inflammatory conditions are last in development, at least in the liver.

In summary, daily exogenous insulin administration caused a progressive deterioration of hepatic insulin signaling. At 15 days of the administration, rats develop reduced glucose tolerance, low HDL levels with FFA increase, and reduced hepatic insulin sensitivity, with a hepatic pro-oxidative microenvironmental, increased MT, and MAPK-ERK1/2 response. At 30 days of the evaluation, hyperinsulinemia with glucose intolerance, low HDL levels with FFA increase, peripheral IR, and low hepatic sensitivity to the hormone were shown. In addition, immunoreactivity increased on insulin receptor (Y1361), IRS (S307), GSK3β (S9), S6K1 (T389), and the number of positive cells to SREBP1c and ChREBP, which results in reduced hepatic glycogen levels and triglyceride accumulation. An increase in the ROS level with MAPK-ERK1/2 response and pro-oxidative microenvironmental was sustained by MT, GSH, and GR activity. Consecutively, at 45 days, the features of prior evaluation are accentuated, hepatic IR appears, MAPK-p38 and profibrotic findings are added, and rats show dyslipidemia. Finally, at 60 days of assessment, the features of prior evaluation are more evident. Zoometric changes were documented (BMI and fat percentage augmented), and an increase in NO_2_^−^ concentration, MAPK-p38, NF-κB, and ApoB was shown. Exogenous insulin administration for extended periods aggravates IR in multiple tissues caused by metabolic, hormonal, oxidative, and inflammatory impairment (data no-show). Thus, insulin signaling is influenced by numerous variables, which could be a disadvantage of the model when studying the influence of oxidative stress and inflammation on hormone signaling in the early stages.

## 5. Conclusions

In conclusion, daily insulin glargine administration (4 U/kg) develops a progressive insulin action impairment time-dependent model, observing significant changes in signaling, glucose and metabolic lipids, redox balance, and zoometry associated with hyperinsulinemia, reduced hepatic insulin sensitivity, peripheral IR, and early hepatic IR. In this IR model, hepatic inflammation did not develop in the early stages.

## Figures and Tables

**Table 1:** Zoometry, oral glucose tolerance, insulin response, resistance, and sensibility indexes.

Parameter	15 Days		30 Days		45 Days		60 Days	
	Control	Insulin	Control	Insulin	Control	Insulin	Control	Insulin
Weight (g)	167 ± 5.83	162.8 ± 2.4	192.6 ± 6.6	205.8 ± 8.3	250 ± 10	251 ± 6	287 ± 15	291.4 ± 16
Size (cm)	15.6 ± 0.19	15.3 ± 0.20	17.4 ± 0.3	18.1 ± 0.4	19.4 ± 0.24	19.4 ± 0.5	21.2 ± 4.2	20.3 ± 1
BMI	0.69 ± 0.01	0.68 ± 0.02	0.64 ± 0.01	0.62 ± 0.02	0.67 ± 0.04	0.67 ± 0.02	0.63 ± 0.1	0.68 ±0.4 *
% Fat	34.6 ± 0.21	34.2 ± 0.4	32.6 ± 0.4	31.7 ± 0.51	31.9 ± 0.6	31.9 ± 0.5	24.3 ± 6	32.6 ± 2 *
Fasting glucose (mg/dL)	98.5 ± 5.1	116 ± 6.5 *	93 ± 3.9	117 ± 4.1 *	97 ± 5.1	131.6 ± 4.7 *	93.6 ± 6.8	112 ± 5 *
Glucose 30′ (mg/dL)	154.5 ± 4.3	164.6 ± 3.9	114.4 ± 5.1	143.5 ± 4.9	113 ± 4.3	159 ± 3.9 *	119.5 ± 6.5	167.5 ± 4.5 *
Glucose 60′ (mg/dL)	165.7 ± 5.5	177.2 ± 6.8	89.6 ± 4	148 ± 2.1 *	133.3 ± 4.6	164.5 ± 4.1 *	117.7 ± 3.3	141 ± 10 *
Glucose 90′ (mg/dL)	179.3 ± 4.2	200.5 ± 5.1 *	97 ± 5	126.5 ± 4.4 *	108.3 ± 2.3	162.4 ± 5.2 *	115 ± 8	188.5 ± 5.5 *
Glucose AUC	13,775	15,020	8,970	12,398 *	10,470	14,118 *	10,245	14,065 *
Insulin fasting (µUI/mL)	43.3 ± 3.4	51.0 ± 4.5	12.5 ± 1.6	16.5 ± 2.2	12.2 ± 1.2	14.1 ± 2.4	9.7 ± 1	12.2 ± 0.6 *
Insulin 30′ (µUI/mL)	52.1 ± 4.1	56.3 ± 2.5	13.1 ± 2.5	15.6 ± 2.1	19 ± 2.2	20.2 ± 1.9	11.7 ± 1.7	23.8 ± 0.7 *
Insulin 60′ (µUI/mL)	64.8 ± 3.8	67.5 ± 4.0	12.1 ± 2.3	20.1 ± 3.4*	17.1 ± 1.5	28.1 ± 2.3 *	11.5 ± 1	33.8 ± 1.3 *
Insulin 90′ (µUI/mL)	69.7 ± 5.1	72.5 ± 3.4	9.2 ± 1.5	22.5 ± 1.1 *	13.1 ± 2.3	18.5 ± 2.4 *	10.3 ± 0.6	23.8 ± 0.9 *
Insulin AUC	5205	5291	1112	1635 *	1469	1694 *	1000	1762 *
HOMA-IR	1.0 ± 0.04	1.6 ± 0.07	0.45 ± 0.01	0.73 ± 0.04 *	0.49 ± 0.03	0.68 ± 0.08 *	0.36 ± 0.02	0.58 ± 0.01 *
QUICKY	0.28 ± 01	0.26 ± 0.06	0.40 ± 0.03	0.31 ± 0.001 *	0.38 ± 0.04	0.30 ± 0.01 *	0.36 ± 0.07	0.32 ± 0.01 *
Matsuda-DeFronzo	1.6 ± 0.06	1.35 ± 0.06	1.3 ± 0.02	0.90 ± 0.08 *	6.63 ± 0.03	4.04 ± 0.19 *	7.61 ± 1.1	4.26 ± 0.1 *
HIS	4.25 ± 0.26	2.71 ± 0.32 *	16.6 ± 0.84	10.21 ± 0.6 *	15.3 ± 0.9	11.2 ± 1 *	16.5 ± 3.1	10.1 ± 0.4 *
LIRI	1.1 ± 0.08	1.3 ± 0.08	0.28 ± 0.13	0.48 ± 0.05	0.24 ± 0.2	0.39 ± 0.01*	0.2 ± 0.02	0.41 ± 0.02 *

The results shown are the average of 10 different experiments ± SEM. (*) indicates significant difference regarding control groups *p* ≤ 0.05 by two-way ANOVA and Bonferroni test. BMI, body mass index; AUC, area under curve; HOMA-IR, homeostasis model assessment insulin resistance; QUICKI, quantitative insulin sensitivity check index; HIS, hepatic insulin sensitivity; LIRI, liver insulin resistance Index.

**Table 2:** Serum lipid profile.

Parameter	15 Days		30 Days		45 Days		60 Days	
	Control	Insulin	Control	Insulin	Control	Insulin	Control	Insulin
Triglycerides	77 ± 2.37	51.2 ± 1.59 *	95.3 ± 2.2	68.2 ± 3.5 *	100 ± 4.3	125 ± 6.7 *	97 ± 4.8	123 ± 4.9 *
VLDL	15.4 ± 0.47	10.2 ± 0.32 *	19 ± 0.4	13.6 ± 0.71 *	21.5 ± 0.9	25 ± 1.3 *	19.4 ± 0.9	24 ± 1.1 *
ApoB	10.5± 1.70	9.8± 1.16	9.7 ± 1	8.3 ± 0.8	11.6 ± 1	10.6 ± 1.5	10.9 ± 0.96	13.1 ±0.8 *
HDL	42.8 ± 1.1	32.8 ± 2.8 *	65 ± 2.2	55 ± 1.5 *	70.2 ± 2	63.7 ± 2.8 *	50.6 ± 1.2	43 ± 2 *
FFA	7.8 ± 0.33	10.4 ± 0.49 *	9.1 ± 0.08	11.6 ± 0.4 *	7.1 ± 0.5	7.7 ± 0.2 *	9.9 ± 0.2	11.8 ± 0.24 *

The results shown are the average of 10 different experiments ± SEM. (*) indicates significant difference regarding control groups *p* ≤ 0.05 by Two-way ANOVA and Bonferroni test. VLDL, very-low-density lipoprotein; ApoB, apoprotein B; HDL, high-density lipoprotein; FFA, free fatty acid.

**Table 3:** Hepatic antioxidant defense.

	15 Days		30 Days		45 Days		60 Days	
	Control	Insulin	Control	Insulin	Control	Insulin	Control	Insulin
Total Glutathione (μM/mg of protein)	19.1 ± 2.2	15.4 ± 2.1	18.9 ± 2.5	15.0 ± 1.4	18.5 ± 2.4	16.7 ± 1.9	18.9 ± 1.2	20.3 ± 1.4
GSH (μM/mg of protein)	13.0 ± 1.3	2.5 ± 0.7 *	12.6 ± 1.8	2.7 ± 1.5 *	13.3 ± 1.7	5.5 ± 1.4 *	12.9 ± 1.6	11 ± 1.3
GSSG (μM/mg of protein)	6.2 ± 0.8	14.8 ± 1.0 *	6.3 ± 0.6	12.7 ± 1.5 *	6.8 ± 0.9	11.6 ± 1.1 *	6.5 ± 1.1	10.3 ± 1.7 *
2GSH/GSSG	4.2 ± 1.0	0.3 ± 0.09 *	4.0 ± 0.5	0.4 ± 0.02 *	4.1 ± 0.4	0.6 ± 0.9 *	4.2 ± 0.8	2.2 ± 0.7 *
GPx (U min^−1^/mg of protein)	2.6 ± 0.1	3.1 ± 0.1	2.3 ± 0.1	2.9 ± 0.2	3.1 ± 0.4	3.2 ± 0.8	2.9 ± 0.2	3.3 ± 0.4
GR (U min^−1^/mg of protein)	1016.2 ± 77	1555.9 ± 160 *	1147.7 ± 170	1759.6 ± 218 *	1051 ± 134	1654 ± 121 *	1152 ± 181	1893 ± 162 *
GT (U min^−1^/mg of protein)	839.9 ± 81	988.1 ± 123	658.2 ± 92	958.7 ± 126	758.2 ± 140	896.4 ± 132	792 ± 164	784.4 ± 170
SOD (U min^−1^/mg of protein)	8.0 ± 0.4	7.1 ± 0.5	7.6 ± 0.9	7.2 ± 0.5	7.7 ± 0.3	7.5 ± 0.4	7.9 ± 0.9	7.3 ± 1.4
CAT (U min^−1^/mg of protein)	19.9 ± 2.4	18.0 ± 2.3	19.5 ± 1.8	18.4 ± 2.4	19.1 ± 1.9	18.5 ± 1.3	20.1 ± 1.4	19.7 ± 2.3
MT (μg/mg of protein)	1.84 ± 0.2	2.4 ± 0.3*	1.3 ± 0.3	2.5 ± 0.5 *	1.9 ± 0.3	2.7 ± 0.7*	1.7 ± 0.4	2.6 ± 0.2 *

The results shown are the average of 10 different experiments ± SEM. (*) indicates significant difference regarding control groups *p* ≤ 0.05 by Student’s *t*-test. GSH, reduced glutathione; GSSG, oxidized glutathione; GPx, glutathione peroxidase; GR, glutathione reductase; GT, glutathione transferase; SOD, superoxide dismutase; CAT, catalase; MT, metallothionein.

## Data Availability

The authors confirm that the majority of the data supporting the findings of this study are available within the article. Raw data are available from the authors upon reasonable request. The data are not publicly available due to privacy restrictions.

## Supplementary Materials

The following supporting information can be downloaded at: https://www.mdpi.com/article/10.3390/metabo13040572/s1, Figure S1: Tissues-Specific Insulin Sensitivity and Resistance Indexes.
