# Supplementary material for: Hepatic Insulin Resistance Model in the Male Wistar Rat Using Exogenous Insulin Glargine Administration
Source: Metabolites. 2023 Apr 18;13(4):572. doi: 10.3390/metabo13040572 (PMC10144445; doi:10.3390/metabo13040572)
Supplement: Supplementary file 1 [file metabolites-13-00572-s001.zip › metabolites-2244987-supplementary.pdf]

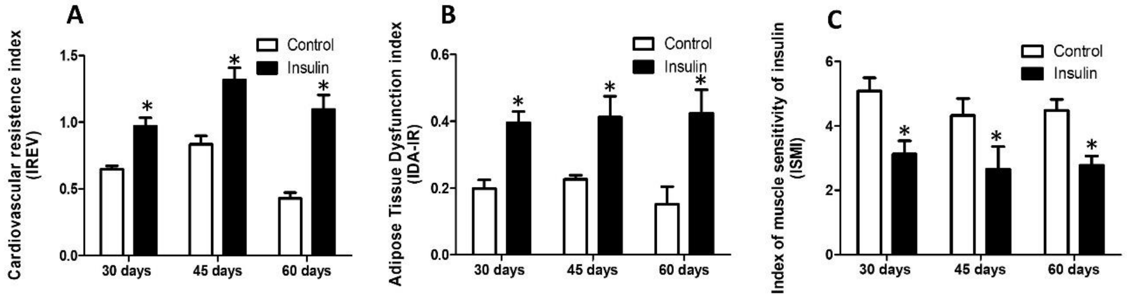

Figure S1. Tissues-Specific Insulin Sensitivity and Resistance Indexes. A) ISMI. **B)** IDA-IR. **C)** IRIEV). The results shown are the average of 10 different experiments ± SEM. A and C graphs were analyzed by a Two-way ANOVA followed by a Bonferroni test. (*) Indicates a significant difference regarding the control group. **ISMI,** Muscle insulin sensitivity index; **IDA-IR**, Insulin resistance adipocyte dysfunction; **IRCV**, Index Cardiovascular Insulin Resistance.
